# Anti-Amyloidogenic Effects of Asarone Derivatives From *Perilla frutescens* Leaves Against Beta-Amyloid Aggregation and Nitric Oxide Production

**DOI:** 10.3390/molecules24234297

**Published:** 2019-11-25

**Authors:** Jae Eun Lee, Nayeon Kim, Ji Yun Yeo, Dae-Gun Seo, Sunggun Kim, Jae-Sun Lee, Kwang Woo Hwang, So-Young Park

**Affiliations:** 1 College of Pharmacy, Dankook University, 119 Dandae-ro, Dongnam-gu, Cheonan-si, Chungnam 31116, Korea; jnlee88@korea.kr (J.E.L.); nayeon02200@gmail.com (N.K.); dankook_jiyun@naver.com (J.Y.Y.); gun3691@naver.com (D.-G.S.); jejui@hanmail.net (S.K.); sailious34@naver.com (J.-S.L.); 2 College of Pharmacy, Chung-Ang University, 84 Heukseok-ro, Dongjak-gu, Seoul 06974, Korea; khwang@cau.ac.kr

**Keywords:** *Perilla frutescens*, asarone derivative, Alzheimer’s disease, beta-amyloid aggregation, beta-amyloid disaggregation, nitric oxide

## Abstract

Alzheimer’s disease (AD) is a progressive, neurodegenerative brain disorder associated with loss of memory and cognitive function. Beta-amyloid (Aβ) aggregates, in particular, are known to be highly neurotoxic and lead to neurodegeneration. Therefore, blockade or reduction of Aβ aggregation is a promising therapeutic approach in AD. We have previously reported an inhibitory effect of the methanol extract of *Perilla frutescens* (L.) Britton (Lamiaceae) and its hexane fraction on Aβ aggregation. Here, the hexane fraction of *P. frutescens* was subjected to diverse column chromatography based on activity-guided isolation methodology. This approach identified five asarone derivatives including 2,3-dimethoxy-5-(1*E*)-1-propen-1-yl-phenol (**1**), β-asarone (**2**), 3-(2,4,5-trimethoxyphenyl)-(2*E*)-2-propen-1-ol (**3**), asaronealdehyde (**4**), and α-asarone (**5**). All five asarone derivatives efficiently reduced the aggregation of Aβ and disaggregated preformed Aβ aggregates in a dose-dependent manner as determined by a Thioflavin T (ThT) fluorescence assay. Furthermore, asarone derivatives protected PC12 cells from Aβ aggregate-induced toxicity by reducing the aggregation of Aβ, and significantly reduced NO production from LPS-stimulated BV2 microglial cells. Taken together, these results suggest that asarone derivatives derived from *P. frutescens* are neuroprotective and have the prophylactic and therapeutic potential in AD.

## 1. Introduction

Alzheimer’s disease (AD) is a chronic, progressive neurodegenerative brain disorder associated with declining memory and cognitive function, occurring in middle or late life [1]. The etiology of AD is poorly understood, many experts suggest that the disease results from a complex interplay of multiple factors rather than any one overriding cause. The predominant “amyloid cascade” hypothesis for AD development proposes that extracellular amyloid β-peptide (Aβ) deposits are fundamental to the pathology of AD [2].

Aβ has been shown to be a major component of the senile plaques of AD patients. The amyloid precursor protein (APP) found on chromosome 21 is cleaved by proteases, β-secretase (BACE1) and γ-secretase to form the Aβ peptide. Aβ is very prone to aggregation, and this process is key to subsequent AD-related pathologies including tau phosphorylation, neurotoxicity, and clinical dementia [3,4]. Additionally, Aβ peptides can stimulate glial cells to produce cytotoxic molecules such as nitric oxide (NO), reactive oxygen species (ROS), and other pro-inflammatory cytokines which significantly contribute to neuronal damage and death [5,6]. Therefore, blockade or reduction of Aβ aggregation is a promising therapeutic approach.

*Perilla frutescens* (L.) Britton var. *acuta* Kudo is a perennial member of the Lamiaceae or mint family. It is primarily cultivated in China, Japan, India, Korea, and other Asian countries. It has been used as a traditional medicine to treat inflammatory diseases, depression, and anxiety-related disorders [7]. Many constituents have been isolated from the *P. frutescens* including rosmarinic acid, caffeic acid, luteolin, elemicin, and apigenin. Ethanol extracts of *P. frutescens* appeared to have strong anti-inflammatory and antioxidant effects [8]. Previous studies have explored the biological activity of specific compounds isolated from *P. frutescens*. Rosmarinic acid was shown to be an antioxidant with anti-allergic and anti-carcinogenic effects [9,10]. Luteolin was shown to have anti-inflammatory, anti-allergic, and anti-tumor effects [11,12], while apigenin alleviated depression [13].

The possible beneficial effects of *P. frutescens* compounds in AD remain poorly understood. Luteolin and rosmarinic acid derived from the methanolic extract of *P. frutescens* have been suggested to act as a β-secretase inhibitor by binding to either a β-secretase subsite or another regulatory site [14]. A previous study in our laboratory described the inhibitory activity of *P. frutescens* against Aβ aggregation, in particular, the methanol extract [15]. Therefore, the purpose of this study was to isolate the active compounds from *P. frutescens* responsible for the observed inhibitory effect on Aβ aggregation and neuroinflammation. A Thioflavin T (ThT) fluorescence assay was performed to determine the levels of Aβ aggregation. A metabolic viability assay using the dye MTT was used to measure the inhibitory effect of isolated compounds on Aβ aggregate-induced toxicity. Additionally, a nitric oxide (NO) release assay was performed to examine the anti-inflammatory effects of *P. frutescens* compounds on LPS-stimulated BV2 mouse microglial cells.

## 2. Results

### 2.1. Isolation and Characterization of the Active Constituents Inhibiting Aβ Aggregation

**Figure 1:**
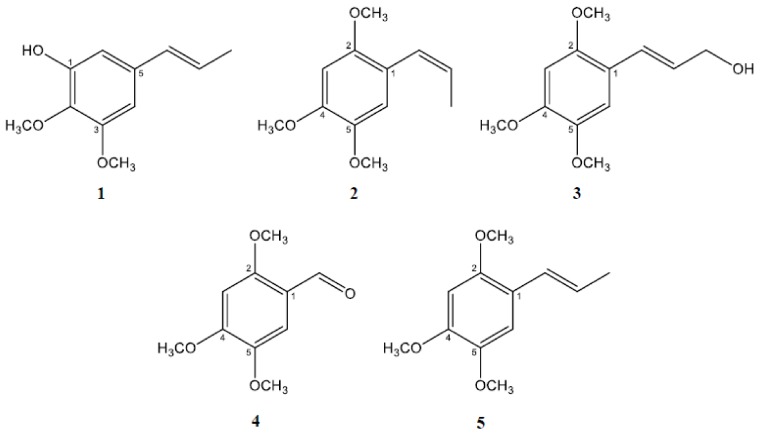
The chemical structures of asarone derivatives. Five asarone derivatives were isolated from *P. frutescens* based on activity-guided isolation methodology; 2,3-dimethoxy-5-(1*E*)-1-propen-1-yl-phenol (**1**), β-asarone (**2**), 3-(2,4,5-trimethoxyphenyl)-(2*E*)-2-propen-1-ol (**3**), asaronealdehyde (**4**), and α-asarone (**5**).

We have previously reported the anti-amyloidogenic effects of *P. frutescens* methanol extract and its hexane fraction [15]. In order to isolate the active constituents of these extracts responsible for inhibiting Aβ aggregation, the hexane fraction of *P. frutescens* was subjected to diverse column chromatographic separation using silica-gel, Sephadex LH-20, and C18 as stationary phases to isolate the active compounds based on the bioassay-guided isolation method. As a result, five asarone derivatives (Figure 1) were isolated as pure compounds including 2,3-dimethoxy-5-(1*E*)-1-propen-1-yl-phenol (**1**) [16], β-asarone (**2**) [17], 3-(2,4,5-trimethoxyphenyl)-(2*E*)-2-propen-1-ol (α-asaryl alcohol, **3**) [18], asaronealdehyde (**4**) [19], and α-asarone (**5**) [20]. The structures of these compounds were determined based on NMR and MS data in comparison with previously reported data.

### 2.2. Asarone Derivatives Inhibit Aβ Aggregation

**Figure 2:**
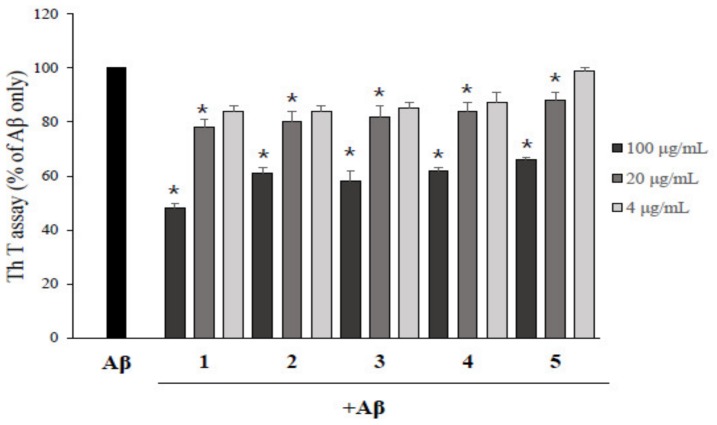
The inhibitory effect of asarone derivatives on Aβ aggregation. The effect of asarone derivatives at 4, 20, and 100 μg/mL on the aggregation of Aβ was determined by ThT assay. Aβ treated with DMSO was used as a control and each experiment was repeated three times. * *p* < 0.05 compared to the Aβ only-treated group.

To determine the inhibition activity of each asarone derivative isolated from *P. frutescens* on Aβ aggregation, a ThT fluorescence assay was performed with DMSO-treated control group. As a control experiment, asarone derivatives were incubated with ThT without Aβ and the the fluorescence values of asarone derivatives with ThT were not significantly different from ThT only (Supplementary Figure S1). As shown in Figure 2, all five asarone compounds inhibited the aggregation of Aβ in a dose-dependent manner. 2,3-Dimethoxy-5-(1*E*)-1-propen-1-yl-phenol (**1**) showed the highest activity, reducing Aβ aggregation to 49.7% at a concentration of 100 µg/mL compared to Aβ alone. β-Asarone (**2**), 3-(2,4,5-trimethoxyphenyl)-(2*E*)-2-propen-1-ol (**3**), asaronealdehyde (**4**), and α-asarone (**5**) were also reduced the Aβ aggregation to 57.5%, 54.8%, 60.2%, and 67.3%, respectively, at the same concentration. These results were accompanied with the western blot analysis with native gels using anti-Aβ(1–20) antibody (Supplementary Figure S2).

### 2.3. Asarone Derivatives Increase the Disaggregation of Pre-Aggregated Aβ

**Figure 3:**
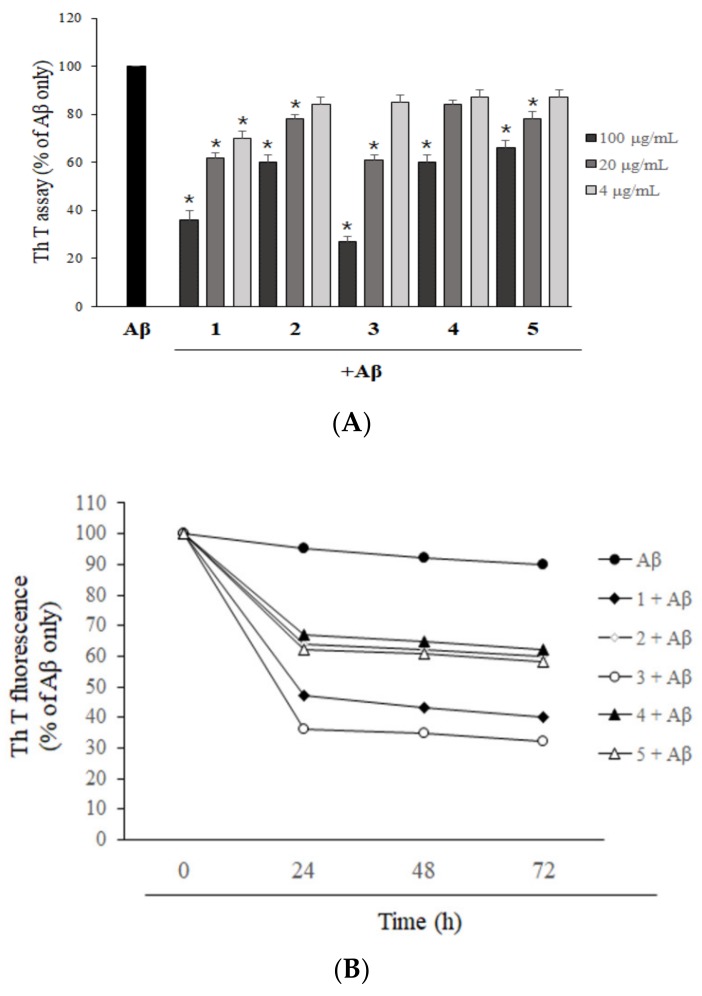
The effect of asarone derivatives on Aβ disaggregation. (**A**) The Aβ that had been pre-aggregated for 24 h was incubated with asarone derivatives for an additional 24 h. A ThT assay was then performed to measure the levels of Aβ aggregation. Aβ-treated with DMSO was used as a control and each experiment was repeated three times. * *p* < 0.05 compared to the Aβ only-treated group. (**B**) Pre-aggregated Aβ was incubated for 24, 48, and 72 h with asarone derivatives and a ThT assay was performed.

To evaluate the effects of isolated asarone derivatives on pre-formed Aβ aggregates, Aβ was aggregated before the addition of asarone derivatives. The degree of Aβ aggregation was then determined using a ThT fluorescence assay. All five asarone derivatives efficiently reduced the levels of Aβ aggregation in a dose-dependent manner (Figure 3A), suggesting that the asarone derivatives are able to disrupt Aβ oligomers. 2,3-Dimethoxy-5-(1*E*)-1-propen-1-yl-phenol (**1**) and 3-(2,4,5-trimethoxyphenyl)-(2*E*)-2-propen-1-ol (**3**) were significantly effective, reducing the level of Aβ aggregation compared to DMSO-treated controls to 34.4% and 24.5%, respectively, at a concentration of 100 µg/mL, and 61.7% and 58.5%, respectively, at 20 µg/mL. However, β-asarone (**2**), asaronealdehyde (**4**), and α-asarone (**5**) exhibited relatively moderate activity in terms of Aβ disaggregation. The time-dependent effect of asarone derivatives on disaggregation of pre-aggregated Aβ was determined by a ThT assay (Figure 3B). Pre-aggregated Aβ for 24 h was further incubated with asarone derivatives for 24, 48, and 72 h. As a result, Aβ alone incubated group showed gradual decrease in the ThT fluorescence during the experiments, but the reduction of the ThT fluorescence was not significant. On the other hand, the ThT fluorescences of Aβ incubated with asarone derivatives were dramatically decreased at 24 h. However, changes in the fluorescence levels were not significantly altered after that up to 72 h.

### 2.4. Asarone Derivatives Protect PC12 Cells from Aβ-Induced Toxicity

**Figure 4:**
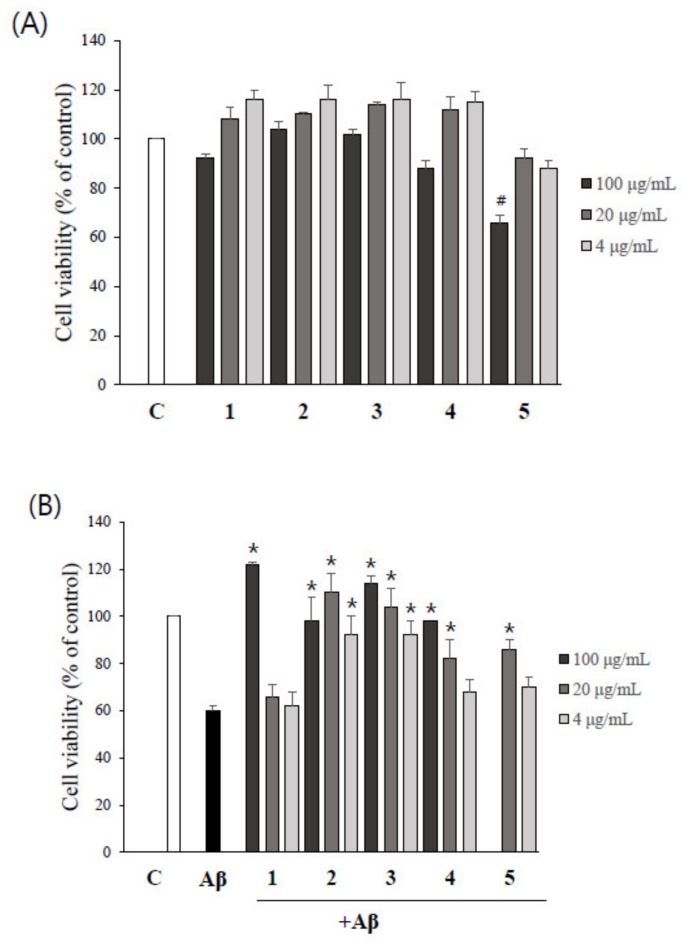
The protective effect of asarone derivatives against Aβ toxicity itself. (**A**) PC12 cells were treated with asarone derivatives for 24 h, and an MTT assay was performed to measure resulting cytotoxicity. DMSO-treated cells were used as a control. ^#^ *p* < 0.05, compared to DMSO-treated control group. (**B**) PC12 cells were pretreated with asarone derivatives for 1 h, then incubated with Aβ in order to evaluate the compounds’ ability to protect the cells from Aβ toxicity itself. Each experiment was repeated at least three times. * *p* < 0.05, compared to the Aβ only-treated group.

The possible cytotoxicity of asarone derivatives themselves on PC12 cells was determined by MTT assay. As shown in Figure 4A, α-asarone (**5**) at 100 µg/mL significantly reduced the viability of PC12 cells, whereas no cytotoxicity was observed at 20 and 4 µg/mL. The remaining four compounds were not cytotoxic to PC12 cells at any of the concentrations tested. Thus, the 100 µg/mL concentration of α-asarone (**5**) was excluded from the further experiments.

To evaluate the possible protective effects of asarone derivatives against Aβ-induced toxicity, PC12 cells were pretreated with asarone derivatives for 1 h, followed by the incubation with Aβ. Treatment of cells with Aβ alone significantly reduced the viability of PC12 cells to 60.2% compared to the DMSO-treated control group. The addition of as little as 4 µg/mL of β-asarone (**2**) and 3-(2,4,5-trimethoxyphenyl)-(2*E*)-2-propen-1-ol (**3**) significantly attenuated Aβ-induced toxicity, resembling the level observed in the DMSO-treated control group. Higher concentrations of 100 µg/mL of 2,3-dimethoxy-5-(1*E*)-1-propen-1-yl-phenol (**1**) and asaronealdehyde (**4**), and 20 µg/mL of α-asarone (**5**) also significantly reduced Aβ-induced toxicity in PC12 cells (Figure 4B).

### 2.5. The Inhibition of Aβ Aggregation by Asarone Derivatives Rescues PC12 Cells from Aβ Toxicity

**Figure 5:**
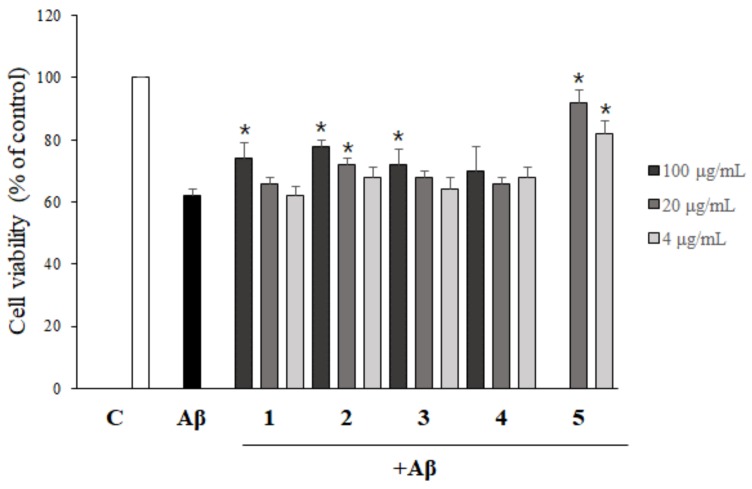
The neuroprotective effect of asarone derivatives on Aβ aggregate-induced toxicity due to inhibition of Aβ aggregation. Aβ monomers were incubated with asarone derivatives for 24 h in order to inhibit Aβ aggregation. This mixture was then added to PC12 cells for an additional 24 h. The change of the cell viability was determined by an MTT assay. Each experiment was repeated at least three times. * *p* < 0.05, compared to the Aβ only-treated group.

In order to evaluate whether the inhibition of Aβ aggregation by asarone derivatives shown in Figure 2 could rescue PC12 cells from Aβ aggregate-induced toxicity, Aβ was incubated with asarone derivatives for 24 h before addition to cells. As shown in Figure 5, 100 and 20 µg/mL of β-asarone (**2**) significantly increased the viability of PC12 cells compared to treatment with Aβ alone. In addition, 20 and 4 µg/mL of α-asarone (**5**) efficiently protected the cells against Aβ aggregate-induced toxicity, resulting in cell viability of 85.7% and 76.2%, respectively. 2,3-Dimethoxy-5-(1*E*)-1-propen-1-yl-phenol (**1**) and 3-(2,4,5-trimethoxyphenyl)-(2*E*)-2-propen-1-ol (**3**) at 100 µg/mL also significantly reduced the cytotoxicity of Aβ aggregate on PC12 cells.

### 2.6. Asarone Derivatives Reduce NO Production in LPS-Stimulated Microglial Cells

**Figure 6:**
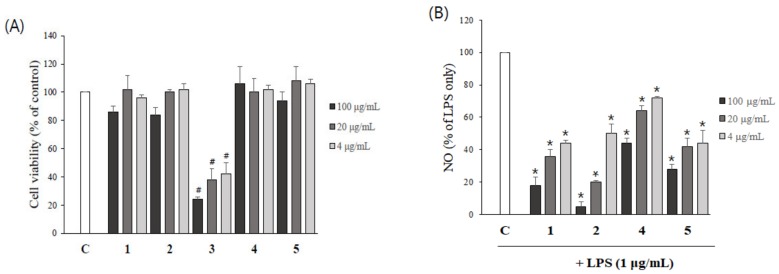
The inhibitory effect of the asarone derivatives on LPS-induced NO production by BV2 cells. (**A**) The cytotoxicity of asarone derivatives on BV2 microglial cells was determined using an MTT assay. ^#^ *p* < 0.05, compared to the DMSO-treated control group. (**B**) BV2 cells pretreated with asarone derivatives for 1 h were incubated with LPS for 24 h, and the production of NO was determined using Griess reagents. Each experiment was repeated at least three times. * *p* < 0.05, compared to the LPS only-treated group.

To evaluate the possible cytotoxicity of asarone derivatives on BV2 microglial cells, cells were treated with various concentrations of asarone derivatives and then MTT assays were performed. With the exception of 3-(2,4,5-trimethoxyphenyl)-(2*E*)-2-propen-1-ol (**3**), no compounds had an effect on the viability of BV2 cells within the concentrations tested (up to 20 µg/mL) (Figure 6A). 3-(2,4,5-Trimethoxyphenyl)-(2*E*)-2-propen-1-ol (**3**) was excluded from the following study.

We then measured the effect of asarone derivatives on LPS-induced NO production by BV2 cells. All the compounds significantly reduced LPS-stimulated NO production in a dose-dependent manner (Figure 6B). 2,3-Dimethoxy-5-(1*E*)-1-propen-1-yl-phenol (**1**), β-asarone (**2**) and α-asarone (**5**) at 5 µg/mL decreased NO production to approximately half the level observed in the LPS-treated group. 20 µg/mL of 2,3-Dimethoxy-5-(1*E*)-1-propen-1-yl-phenol (**1**) and β-asarone (**2**) were particularly effective, reducing NO production to 16.3% and 4.9%, respectively, compared to LPS alone.

## 3. Discussion

Senile plaques composed of Aβ peptides, one of the pathologic hallmarks of AD, are generated by proteolytic fragmentation of APPs. Many studies have implicated that Aβ aggregates, rather than the monomer form, as a neurotoxic agent that leads to neurodegeneration in AD [21]. Therefore, blockade or reduction of Aβ aggregation is a promising therapeutic approach for AD [22,23].

Natural products and their active constituents have been suggested to be beneficial for treating neurodegenerative diseases including AD. For example, galantamine isolated from snowdrop has been approved by the FDA for the treatment of AD as an inhibitor of acetylcholine esterase [24,25]. Curcumin derivatives have been shown to possess a wide range of beneficial biological activities against AD as inhibitors of Aβ aggregation [26,27]. Rosmarinic acid was also reported to have neuroprotective, antioxidative, and anti-amyloidogenic properties [28]. Biflavonoids from *Garcinia madruno* exhibited beneficial effects in a transgenic mouse model of AD [29]. Crocin from *Crocus sativus* [30], and phenylpropanoids and lignans from the seeds of *Prunus tomentosa* [31] were shown to have anti-Aβ aggregation activity. While many studies have reported an inhibitory effect of compounds derived from natural products on Aβ aggregation, few have identified both anti-Aβ aggregation and Aβ disaggregation properties. Compounds with the capacity to inhibit Aβ aggregation, as well as disaggregate preformed Aβ aggregate could be an attractive candidate for development as novel therapeutic agents for AD.

In this study, five asarone derivatives including 2,3-dimethoxy-5-(1*E*)-1-propen-1-yl-phenol (**1**), β-asarone (**2**), 3-(2,4,5-trimethoxyphenyl)-(2*E*)-2-propen-1-ol (**3**), asaronealdehyde (**4**), and α-asarone (**5**) were isolated as active constituents from *P. frutescens*. To our knowledge, this is the first time 2,3-dimethoxy-5-(1*E*)-1-propen-1-yl-phenol (**1**) has been isolated from *P. frutescens*, and that 3-(2,4,5-trimethoxyphenyl)-(2*E*)-2-propen-1-ol (**3**) has been purified from a biological sample.

Asarone derivatives including α- and β-asarone are major bioactive phytochemicals present in the Acorus species such as *Acorus calamus* Linn and *Acorus gramineus* Solander [32,33], and *Guatteria gaumeri* Greenman [34]. The wide range of pharmacological activities of asarone derivatives have been reported including antiepileptic [35], antidepressant [36], anxiolytic [37], neuroprotective [38], and hypolipidemic activities [39]. Numerous clinical studies in China had reported that asarone derivatives were effective therapeutic approaches for the treatment of respiratory disorders and epilepsy [40,41]. Thus, it is considered to be potentially useful in the treatment of various diseases, particularly CNS disorders. On the other hand, acute and sub-chronic toxicities of α- and β-asarone in pre-clinical toxicological studies were not detected, whereas they might cause hepatocellular carcinoma in chronic toxicity test due to the epoxide metabolites [42,43,44,45]. However, the extensive dose-dependent in vivo toxicological studies are required to confirm the toxicity of asarone derivatives. Therefore, the selection of dose and duration time of asarone derivatives is carefully determined to avoid the toxicity in human studies.

Among the five asarone derivatives we studied, β-asarone (**2**) has been reported to protect PC12 cells against Aβ-induced neurotoxicity [46], and improve learning and memory in APP/PS1 transgenic mice [47]; α-Asarone (**5**) was reported to prevent neuroinflammation by inhibiting NF-κB activation [48]. Nonetheless, this is the first report regarding the effect of asarone derivatives on the inhibition of Aβ aggregation, the enhancement of Aβ disaggregation, and the subsequent protective effects on disease-relevant cell lines.

In this study, five asarone derivatives were isolated from *P. frutescens* based on activity-guided isolation methodology using diverse column chromatography. All five asarone derivatives efficiently reduced the aggregation of Aβ, and furthermore, significantly increased the disaggregation of Aβ aggregate. Consequently, the inhibition of Aβ aggregation by asarone derivatives efficiently rescued the PC12 cells from Aβ aggregate-induced toxicity. In addition, asarone derivatives significantly inhibited LPS-induced NO production by BV2 microglial cells. Taken together, these observations suggest that the asarone derivatives isolated from *P. frutescens* could exert beneficial effects against AD. Therefore, the asarone derivatives and *P. frutescens* have the potential to be developed as therapeutic or preventative drugs for AD.

## 4. Materials and Methods

### 4.1. Chemicals and Reagents

RPMI 1640 was purchased from Welgene (Daegu, Korea). Horse serum was purchased from Gibco BRL (Carlsbad, CA, USA). Fetal bovine serum (FBS) was purchased from Equitech-BIO (Kerrville, TX, USA). Aβ_1–42_ was purchased from GL Biochem (Shanghai, China). Aβ_25–35_ was purchased from Bachem AG (Bubendorf, Switzerland). Thioflavin T, isoproterenol and 3-(4,5-dimethyl-2-thiazolyl)-2,5-diphenyl-2H-tetrazolium bromide (MTT) was purchased from Sigma Aldrich (St. Louis, MO, USA). Dimethyl sulfoxide was purchased from Wako Pure Chemical (Mie, Japan). Acetonitrile for high performance liquid chromatography (HPLC) was purchased from Samchun (Pyeong-taek, Korea). Griess reagent was purchased from Promega Co (Madison, WI, USA).

### 4.2. Plant Material

The leaves of *P. frutescens* were purchased from a commercial market (Samhong medicinal herb market; Seoul, Korea) in 2014. A voucher specimen has been deposited in Pharmacognosy Laboratory of College of Pharmacy, Dankook University, Korea.

### 4.3. Extracts and Isolation

Dried and pulverized *P. frutescens* leaves (5 kg) were extracted with 90% of methanol (MeOH, 40 L, 3 times) at room temperature. The MeOH filtrate was evaporated under vacuum to yield the MeOH extract (308 g). The extract was suspended in distilled water and then partitioned sequentially into *n*-hexane (111.5 g), dichloromethane (5.6 g), ethyl acetate (16.9 g) and water (119 g). Each layer was dissolved in dimethyl sulfoxide (DMSO) for the bioassay.

The *n*-hexane fraction was dried and further fractionated on silica-gel column chromatography with a solvent mixture of *n*-hexane: ethyl acetate (20:1, 10:1, 5:1, 1:1; 2L of each solvent mixture) and seven fractions were obtained (PFH1~PFH 7). Two g of fraction PF3 was chromatographed on a medium pressure liquid chromatography (MPLC, Isolera One, Biotage, Korea) using 50% MeOH isocratic and five fractions were obtained (PFH3-1–PFH3-5). Among five subfractions, PFH3-3 was further chromatographed on a MPLC with same condition, and the subfraction PFH3-3-4 was obtained as a pure compound (compound **1,** 2,3-dimethoxy-5-(1*E*)-1-propen-1-yl-phenol, 22.7 mg). Subfraction PFH3-3-6 was further purified with semi-preparative HPLC (YMC-Pack ODS-A, 250 × 10 mm, 5 μm) using 40% acetonitrile isocratic elution system and compound **2** (β-asarone, 1.0 mg) was obtained. The fraction PFH3-3-2 was purified with semi-preparative HPLC using 20%–100% acetonitrile, compound **3** (3-(2,4,5-trimethoxyphenyl)-(2*E*)-2-propen-1-ol, 0.9 mg) and compound **4** (asaronealdehyde, 6.6 mg) were obtained. The fraction PFH3-5 was obtained as a pure compound (compound **5**, α-asarone, 200 mg).

The purified compounds were subjected to nuclear magnetic resonance (NMR). ^1^H (700 MHz) and ^13^C NMR (177 MHz) were investigated with a NMR spectrometer (AdvanceIII, Bruker, Germany).

*2,3-Dimethoxy-5-[(1E)-1-propen-1-yl]-phenol* (**1**) C_1__1_H_1__4_O_3_ MS *m/z* 195 [M + H]^+^. ^1^H NMR (CD_3_OD) δ: 6.50 (1H, d, *J* = 0.7 Hz, H-4), 6.50 (1H, d, *J* = 0.7 Hz, H-6), 6.27 (1H, dd, *J* = 1.4, 15.4 Hz, H-7), 6.14 (1H, dq, *J* = 7.0, 15.8 Hz, H-8), 1.85 (3H, dd, *J* = 1.4, 6.7 Hz, H-9), 3.78 (3H, s, H-10), 3.83 (3H, s, H-11). ^13^C-NMR (CD_3_OD) δ: 150.1 (C-1), 135.5 (C-2), 153.1 (C-3), 101.3 (C-4), 134.1 (C-5), 106.3 (C-6), 130.8 (C-7), 124.1 (C-8), 17.1 (C-9), 54.9 (C-10), 59.6 (C-11). logP (o/w): 2.370 (est).

*β-asarone* (**2**) C_12_H_16_O_3_ MS *m*/*z* 209 [M + H]^+^. ^1^H NMR (CD_3_OD) δ: 6.68 (1H, s, H-3), 6.88 (1H, s, H-6), 6.44 (1H, dq, *J* = 2.1, 11.6 Hz, H-7), 5.71 (1H, dq, *J* = 7.0, 11.9 Hz, H-8), 1.83 (3H, dd, *J* = 2.1, 7.0 Hz, H-9), 3.88 (3H, s, H-10), 3.81 (3H, s, H-11), 3.80 (3H, s, H-12). ^13^C-NMR (CD_3_OD) δ: 118.2 (C-1), 152.0 (C-2), 97.9 (C-3), 149.0 (C-4), 142.3 (C-5), 114.9 (C-6), 124.7 (C-7), 124.6 (C-8), 13.5 (C-9), 56.2 (C-10), 55.4 (C-11), 55.3 (C-12).logP (o/w): 3.406 (est).

*3-(2,4,5-trimethoxyphenyl)-(2E)-2-Propen-1-ol* (**3**) C_12_H_16_O_3_ MS *m/z* 225 [M + H]^+^. ^1^H NMR (CD_3_OD) δ: 6.66 (1H, s, H-3), 7.08 (1H, s, H-6), 6.85 (1H, dt, *J* = 1.4, 16.1 Hz, H-7), 6.25 (1H, dt, *J* = 6.3, 16.1 Hz, H-8), 4.22 (2H, dd, *J* = 1.4, 6.3 Hz, H-9), 3.88 (3H, s, H-10), 3.84 (3H, s, H-11), 3.82 (3H, s, H-12). ^13^C-NMR (CD_3_OD) δ: 117.7 (C-1), 153.1 (C-2), 97.4 (C-3), 150.3 (C-4), 144.1 (C-5), 110.7 (C-6), 125.8 (C-7), 128.6 (C-8), 62.9 (C-9), 56.0 (C-10), 55.6 (C-11), 55.2 (C-12). logP (o/w): 1.290 (est).

*asaronealdehyde* (**4**) C_10_H_12_O_4_ MS *m*/*z* 197 [M + H]^+^. ^1^H NMR (CD_3_OD) δ: 6.73 (1H, s, H-3), 7.28 (1H, s, H-6), 10.2 (1H, s, H-7), 3.97 (3H, s, H-8), 3.96 (3H, s, H-9), 3.82 (3H, s, H-10). ^13^C-NMR (CD_3_OD) δ: 116.7 (C-1), 159.4 (C-2), 96.3 (C-3), 156.8 (C-4), 143.6 (C-5), 109.0 (C-6), 188.1 (C-7), 55.5 (C-8), 55.4 (C-10), 55.4 (C-11). logP (o/w): 1.634 (est).

*α-asarone* (**5**) C_12_H_16_O_3_ MS *m*/*z* 209 [M + H]^+^. ^1^H NMR (CD_3_OD) δ: 6.62 (1H, s, H-3), 7.01 (1H, s, H-6), 6.11 (1H, dq, *J* = 2.1, 14.0 Hz, H-7), 4.89 (1H, dq, *J* = 6.3, 15.4 Hz, H-8), 1.86 (3H, dd, *J* = 2.1, 6.7 Hz, H-9), 3.85 (3H, s, H-10), 3.81 (3H, s, H-11), 3.80 (3H, s, H-12). ^13^C-NMR (CD_3_OD) δ: 119.0 (C-1), 151.1 (C-2), 98.1 (C-3), 149.0 (C-4), 143.2 (C-5), 110.5 (C-6), 125.0 (C-7), 123.1 (C-8), 17.5 (C-9), 56.0 (C-10), 55.6 (C-11), 55.2 (C-12). logP (o/w): 2.794 (est).

### 4.4. Thioflavin T (ThT) Assay

To evaluate the aggregate formation of Aβ, a ThT assay was performed [15]. The Aβ_1–42_ was dissolved in DMSO at 1 mg/mL concentration and five asarone derivatives were diluted in DMSO. To monitor the effects of asarone derivatives on the aggregate of Aβ, 20 µM of Aβ_1–42_ was incubated together with various concentrations (4, 20, and 100 µg/mL) of asarone derivatives at 37 °C for 24 h. Then, 3 µM of ThT was added (protected from light) and fluorescence was measured after 30 min using an Emax precision microplate reader (black plate) (Molecular Devices, CA, USA) with excitation at 442 nm and emission at 485 nm. The Aβ treated with DMSO was used as a control and each assay was performed in triplicate.

To monitor the disaggregation effects of asarone derivatives on pre-aggregated Aβ, a ThT assay was performed. Briefly, 20 µM of Aβ_1–42_ was incubated at 37 °C for 24 h. Various concentrations (4, 20, and 100 µg/mL) of asarone derivatives were then added for an additional incubation at 37 °C for additional 24 h (protected from light). Then, 3 µM of ThT was added and fluorescence was measured after 30 min using an Emax precision microplate reader (black plate) with excitation at 442 nm and emission at 485 nm. Aβ only-treated group was used as a control and each assay was performed in triplicate.

### 4.5. Cell Viabiltiy Assay

PC12 cells (rat pheochromocytoma cells) were obtained from Korea Cell Line Bank (Seoul, Korea) and grown in RPMI 1640 medium supplemented with 5% heat-inactivated FBS and 15% heat-inactivated horse serum. The cells were incubated in a humidified 5% CO_2_ atmosphere at 37 °C. The cells growing exponentially were harvested from flasks and plated in 96-well plates with approximately 6 × 10^4^cells per 100 µL of medium per well. Plates were incubated at 37 °C for 3 h to allow the cells to attach to the plates.

In order to determine the possible cytotoxicity of asarone derivatives themselves, compounds were added to individual wells (4, 20, and 100 µg/mL). The plates were then incubated for 24 h at 37 °C. Cell viability was determined using a MTT toxicity assay by adding 10 µL of 5 mg/mL MTT to each well. After 3 h of incubation at 37 °C, 100 µL of DMSO was added to each well. Plates were incubated at room temperature for 30 min to dissolve the MTT formazan crystals and the absorbance at 570 nm was measured using a Emax precision microplate reader. Averages from three replicate wells were used for each sample and each assay was performed in triplicate.

In order to determine the protective effect of asarone derivatives against Aβ, the cells in 96-well plates were pre-treated with asarone derivatives (4, 20, and 100 µg/mL) for 1 h and then followed by the treatment with Aβ (Aβ_25–35_, 10 µM). The plates were then incubated for an additional 24 h at 37 °C. Cell viability was determined using a MTT toxicity assay. Averages from three replicate wells were used for each sample and each assay was performed in triplicate.

To investigate whether the inhibition of Aβ aggregation by asarone derivatives affects the viability of PC12 cells against Aβ aggregate-induced toxicity, compounds (4, 20, and 100 µg/mL) and Aβ_1–42_ (10 µM) were mixed together in 96-well plates and incubated at 37 °C for 24 h to inhibit the formation of Aβ aggregates. Then, the mixture was added to the cells in 96-well plates and incubated at 37 °C for an additional 24 h. Cell viability was determined using a MTT toxicity assay. Averages from three replicate wells were used for each sample and each assay was performed in triplicate.

### 4.6. Determination of NO production

BV2 cells (immortalized mouse microglial cells) were grown in a DMEM medium supplemented with 5% heat-inactivated FBS. The cells were incubated in a humidified 5% CO_2_ atmosphere at 37 °C. BV2 cells growing exponentially were harvested from flasks and plated in 96-well plates with approximately 6 × 10^4^cells per 100 µL of medium per well. Plates were incubated at 37 °C for 3 h to allow the cells to attach to the plates. Compounds (4, 20, and 100 µg/mL) were added to individual wells. After 1 h, LPS was added in each culture well with 1 µg/mL of concentration. The plates were then incubated for an additional 24 h at 37 °C. The NO production was determined using Griess reagents [49]. The supernatant was mixed with an equal volume of the sulfanilamide solution and incubated at 37 °C for 10 min in the dark. Then the NED solution was added with same volume and incubated for 10 more min in the dark. The absorbance at 540 nm was measured using a Emax precision microplate reader. Averages from three replicate wells were used for each sample and each assay was performed in triplicate.

### 4.7. Statistical Analyses

All data in the text and figures are expressed as means ± SD. Two or more group comparisons were evaluated by one-way analysis of variance followed by Tukey post hoc text (SPSS version 17.0, Armonk, NY, USA). Differences were considered statistically significant at *p* < 0.05.

## Supplementary Materials

The following are available online at https://www.mdpi.com/1420-3049/24/23/4297/s1, Figure S1: Direct effect of asarone derivatives on Th T fluorescence, Figure S2: The effect of asarone derivatives on Aβ aggregation determined by western blot analysis.
