# Supplementary material for: Anti-Amyloidogenic Effects of Asarone Derivatives From Perilla frutescens Leaves Against Beta-Amyloid Aggregation and Nitric Oxide Production
Source: Molecules. 2019 Nov 25;24(23):4297. doi: 10.3390/molecules24234297 (PMC6930631; doi:10.3390/molecules24234297)
Supplement: Supplementary file 1 [file molecules-24-04297-s001.pdf]

Supplementary Materials

Anti-Amyloidogenic Effects of Asarone Derivatives From *Perilla frutescens* Leaves Against Beta-Amyloid Aggregation and Nitric Oxide Production

Jae Eun Lee [1,†], Nayeon Kim [1,†], Ji Yun Yeo [1], Dae-Gun Seo [1], Sunggun Kim [1], Jae-Sun Lee [1], Kwang Woo Hwang [2] and So-Young Park [1,*]

[1] College of Pharmacy, Dankook University, 119 Dandae-ro, Dongnam-gu, Cheonan-si, Chungnam 31116, Republic of Korea; jnlee88@korea.kr (J.E.L.); nayeon02200@gmail.com (N.K.); dankook_jiyun@naver.com (J.Y.Y.); gun3691@naver.com (D.-G.S.); jejui@hanmail.net (S.K.); sailious34@naver.com (J.-S.L.)

[2] College of Pharmacy, Chung-Ang University, 84 Heukseok-ro, Dongjak-gu, Seoul 06974, Republic of Korea; khwang@cau.ac.kr

† These authors contributed equally to this work.

[*] Correspondence: soypark23@dankook.ac.kr, Tel.: +82-41-550-1434, Fax: +82-41-559-7899

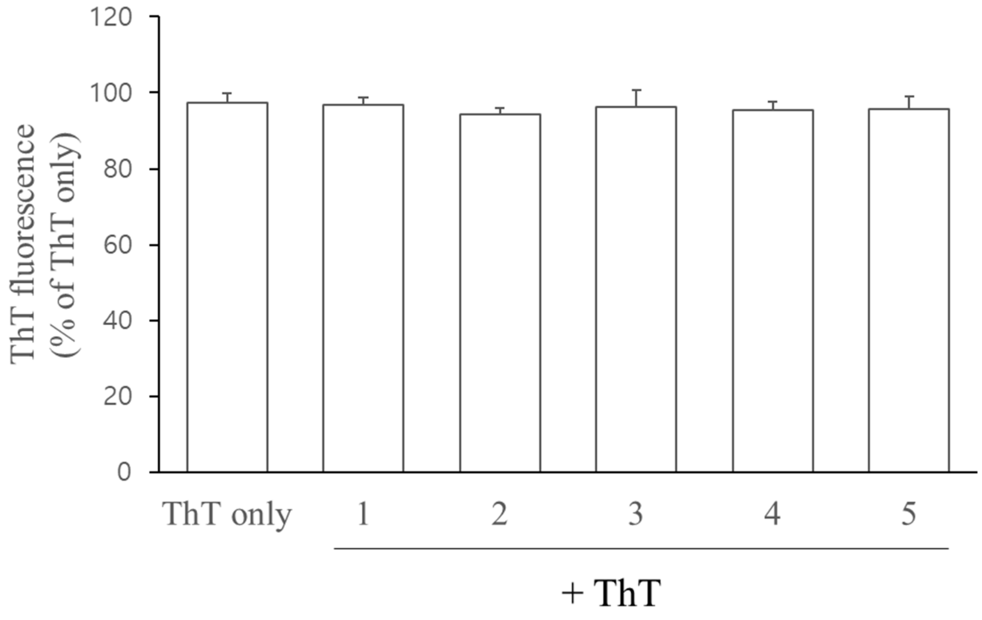

Supplementary Figure 1. Direct effect of asarone derivatives on Th T fluorescence. In order to determine quenching effect of asarone derivatives asarone derivatives were incubated with Th T without Aβ and the fluorescence values were determined. The Th T fluorecence values were compared to Th T only.

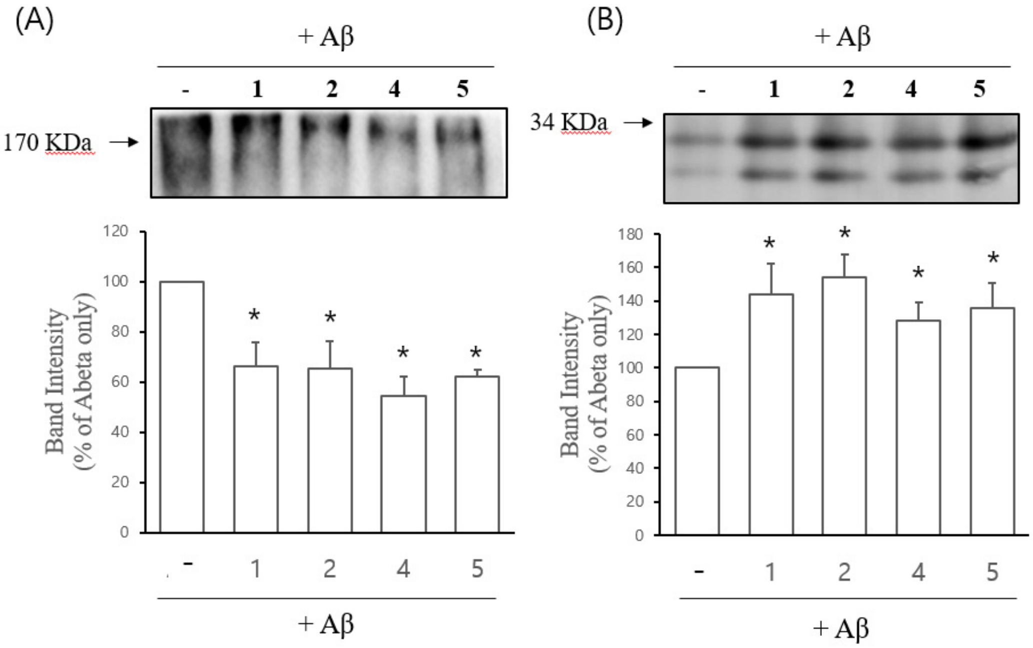

Supplementary Figure 2. The effect of asarone derivatives on Aβ aggregation determined by western blot analysis. The western blot analysis with native gels using anti-Aβ(1–20) antibody and the band intensity was compared to Aβ only samples (Due to the lack of amount, compound **3** was excluded from this experiment). * $p < 0.05$ compared to Aβ only.
